# Morin Hydrate Inhibits TREM-1/TLR4-Mediated Inflammatory Response in Macrophages and Protects Against Carbon Tetrachloride-Induced Acute Liver Injury in Mice

**DOI:** 10.3389/fphar.2019.01089

**Published:** 2019-09-20

**Authors:** Xi Li, Qunyan Yao, Jiying Huang, Qianwen Jin, Beili Xu, Fangyuan Chen, Chuantao Tu

**Affiliations:** ^1^Department of Geriatrics, Zhongshan Hospital, Fudan University, Shanghai, China; ^2^Department of Gastroenterology and Hepatology, Zhongshan Hospital, Fudan University, Shanghai, China; ^3^Department of Gastroenterology and Hepatology, Zhongshan Hospital Qingpu Branch, Fudan University, Shanghai, China

**Keywords:** liver injury, morin hydrate, inflammatory response, TREM-1, TLR4, NF-κB, oxidative stress

## Abstract

This study aims to investigate the protective effects of morin hydrate (MH) against acute liver injury induced by carbon tetrachloride (CCl_4_) in mice and to elucidate the possible molecular mechanism of action. Mice were pretreated with MH (50 mg/kg body weight) or vehicle by oral gavage once daily for 5 days, followed by intraperitoneal injection of a single dose of CCl_4_ (1 ml/kg in olive oil). Mice were sacrificed 24 h later; the blood and liver samples were harvested for analysis. We also used the model of lipopolysaccharide (LPS)-stimulated RAW264.7 macrophages *in vitro* and examined the effects of MH and its mechanism of action on the inflammatory response. Our results revealed that MH remarkably attenuated liver histopathological alterations, serum transaminases, hepatocytes death, and inflammatory response induced by CCl_4_. Importantly, MH reduced expression of the triggering receptor expressed on myeloid cells-1 (TREM-1) and toll-like receptor 4 (TLR4) both *in vivo* and *in vitro* experiments. This inhibitory effect MH on expression of the TREM-1 and TLR4 in cell culture was further heightened after TREM-1 knockdown with small interfering RNA (siRNA). Moreover, MH dramatically suppressed the inhibitor of kappa B α (IκBα) degradation and subsequent nuclear factor-kappa B (NF-κB) p65 translocation into the nucleus and NF-κB-mediated cytokines, such as tumor necrosis factor α (TNF-α), interleukin (IL)-1β, and IL-6. Additionally, MH also ameliorated CCl_4_-induced oxidative stress by enhancing the nuclear factor erythroid 2-related factor 2 (Nrf2) and heme oxygenase-1 (HO-1) expression in the injured livers. Taken together, MH has hepatoprotective activity, and this effect may be elicited by attenuating macrophage-mediated inflammatory responses *via* inhibition TREM-1/TLR4/NF-κB signaling and by regulating hepatic oxidative stress *via* enhancement Nrf2/HO-1 antioxidant pathway.

## Introduction

Acute liver injury is a common pathway to many liver diseases and remains a serious health problem worldwide associated with significant morbidity and mortality (Malhi and Gores, 2008; Bernal et al., 2010). It is worth noticing that if acute liver injury persists, it may lead to chronic liver inflammation and fibrosis, eventually progressing to cirrhosis and hepatocellular carcinoma (Malhi and Gores, 2008; Reilkoff et al., 2011; Dong et al., 2015). However, there is no specific treatment for acute liver injury. Hence, it is urgently needed to develop novel therapeutic methods to prevent acute liver injury and inflammation.

Hepatic inflammation is a complex process that is a response to a wide range of stimuli that induce oxidative stress and injury to hepatocytes (Kubes and Mehal, 2012; Zhang et al., 2015; Li et al., 2016b; Hassan et al., 2017; Krenkel and Tacke, 2017). Previous data have demonstrated that some endogenous and exogenous antioxidants may protect against oxidative-induced liver injury and inflammation (Lu et al., 2012; He et al., 2016; Lin et al., 2018; Mortezaee and Khanlarkhani, 2018). Actually, it has been suggested that, in most cases, hepatocellular injury is not due to the damaging agent itself but to the activation of the immune cells such as Kupffer cells (He et al., 2016; Krenkel and Tacke, 2017; Lin et al., 2018). It is well known that Kupffer cells are liver-resident tissue macrophages and the principal mediators for native and adaptive inflammatory responses (Antoniades et al., 2008; Krenkel and Tacke, 2017). Moreover, several lines of evidence have indicated that inflammatory responses mediated by cytokines and chemokines play an important role in cell death and liver injury (Sims et al., 2010; Kubes and Mehal, 2012; Lu et al., 2012; Krenkel and Tacke, 2017).

Triggering receptor expressed on myeloid cells-1 (TREM-1), a member of TREM family, is a kind of immunoglobulin (Ig) superfamily activation receptor related to innate inflammatory response (Tammaro et al., 2017). Of note, TREM-1 is an activating receptor expressed on neutrophils and monocyte/macrophages (Ornatowska et al., 2007; Tammaro et al., 2017). Upon activation, TREM-1 can trigger and amplify inflammatory responses, especially through interaction with toll-like receptor (TLR) signaling in macrophages (Klesney-Tait et al., 2006; Ornatowska et al., 2007; Tammaro et al., 2017). Previous studies have further demonstrated TREM-1 is a master regulator of Kupffer cell activation, which escalates acute and chronic inflammatory responses in liver diseases (Nguyen-Lefebvre et al., 2018; Rao et al., 2019). A recent study found that knockdown of TREM-1 can ameliorate the inflammatory response and lipid accumulation of nonalcoholic fatty liver disease (NAFLD) mice inactivation of TREM-1/nuclear factor-kappa B (NF-κB) and TREM-1/PI3K/AKT axis (Rao et al., 2019). In addition, previous studies have revealed that TLR-mediated signals are involved in almost all liver diseases (Seki et al., 2007; Mencin et al., 2009; Li et al., 2016a), and TLR4/NF-κB signaling is of particular importance for liver injury (Seki et al., 2007; Li et al., 2016a). NF-κB is a cardinal regulator of inflammatory response, controlling the expression of genes that encode cytokines such as tumor necrosis factor α (TNF-α), interleukin (IL)-1β, and IL-6 (Seki et al., 2007; Mencin et al., 2009; Li et al., 2016a). Therefore, these data indicate that TREM-1/TLR4-mediated inflammatory response plays a pivotal part in liver injury and inflammation and may serve as a promising therapeutic target for preventing acute liver injury.

**Figure 1:**
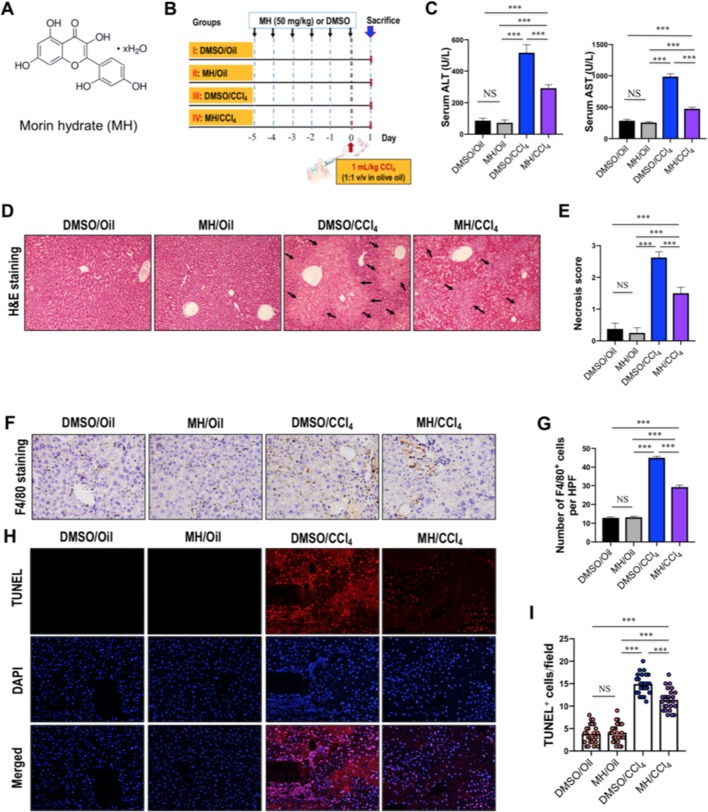
Effect of morin hydrate (MH) on CCl_4_-induced liver injury in mice. **(A)** The molecular structure of MH (2′,3,4′,5,7-pentahydroxyflavone). **(B)** Experimental study design. Mice were randomly divided into four groups, each consisting of eight animals. MH was dissolved in dimethyl sulfoxide (DMSO), and mice were orally given 50 mg/kg of MH once a day for five consecutive days before challenge with CCl_4_. To induce acute liver injury, a single dose of 1 ml/kg CCl_4_ (1:1 v/v in olive oil) was injected intraperitoneally (i.p.) for 24 h. **(C)** Serum alanine transaminase (ALT) and aspartate transaminase (AST) levels in mice from each group. Data are expressed as mean ± SEM (*n* = 6 for each group). **(D)** Histological examination of liver sections from each group with hematoxylin and eosin (H&E) staining (original magnification, ×100). Black arrows indicate area of necrosis. **(E)** Necrosis was analyzed based on H&E-stained liver sections and scored as described in the Material and Methods section (*n* = 8 for each group). **(F)** Immunohistochemical detection of F4/80-positive cells in liver sections from each group (original magnification: ×200). **(G)** Quantification of F4/80-positive cells per high-power field (HPF) in liver sections from each group. Results mean of six fields and *n* = 5 for each group. **(H)** TUNEL staining for apoptotic cells of the liver sections from each group of mice (original magnification, ×200). **(I)** The quantification of TUNEL-positive cell number per HPF (×400). Cells were counted five visual per liver sections from each group and *n* = 5 for each group. ***P < 0.001; NS indicates not significant.

Morin hydrate (MH; 2′,3,4′,5,7-pentahydroxyflavone; **Figure 1A**), a member of the flavonoids, is a yellow-colored compound that can be separated from members of the Moraceae plants family (Lee et al., 2016; Naowaboot et al., 2016); it is also found in many herbs, red wine, and fruits like almond, guava, sweet chestnut, and onion (Caselli et al., 2016; Lee et al., 2016; Naowaboot et al., 2016). MH has been reported to possess antioxidant, neuroprotective, anti-inflammatory, antihypertensive, and anticancer properties (Dong et al., 2015; Caselli et al., 2016; Lee et al., 2016; Naowaboot et al., 2016). In particular, MH has also shown to inhibit liver injury, inflammation, and fibrosis in several different animal models by its antioxidant and anti-inflammatory effects (Rizvi et al., 2015; Gu et al., 2017; Perumal et al., 2017; Tian et al., 2017). However, the exact molecular mechanism underlying the hepatoprotective effect of MH is still partially understood. Therefore, the aims of this study were to investigate the protective effects of MH against acute liver injury in mice and to elucidate its underlying mechanisms.

## Materials and Methods

### Reagents and Antibodies

MH, lipopolysaccharide (LPS), dimethyl sulfoxide (DMSO), and 3-(4,5-dimethylthiazol-2-yl)-2,5-diphenyltetrazolium bromide (MTT) were purchased from Sigma-Aldrich (St. Louis, MO, USA). Carbon tetrachloride (CCl_4_) was purchased from Shanghai Macklin Chemical Co., Ltd. (Shanghai, China). TNF-α and IL-1β enzyme-linked immunosorbent assay (ELISA) kits were from R&D Systems (Minneapolis, MN, USA). Lipid peroxidation malondialdehyde (MDA) assay kit, total glutathione assay kit, and total superoxide dismutase (SOD) assay kit were from Beyotime Institute Biotechnology (Shanghai, China). TRIzol reagent is from Life Technologies (Grand Island, NY, USA). Mouse anti-NFκB p65 polyclonal antibody and rabbit anti-IκB monoclonal antibody were from Cell Signaling Technology (Boston, MA, USA); rabbit anti-TLR4 monoclonal antibody was from Epitomics, Inc. (Burlingame, CA); rabbit anti-Nrf2 monoclonal antibody, rabbit anti-TREM-1 polyclonal antibody, rat anti-F4/80 monoclonal antibody, rabbit anti-HO-1 polyclonal antibody, rabbit anti-GAPDH monoclonal antibody, and rabbit anti-Lamin B1 monoclonal antibody were from Abcam (Cambridge, MA).

### Cell Culture

RAW264.7 murine macrophage cells were obtained from Sigma Chemical, Co. Ltd. (St. Louis, MO) and cultured in undifferentiated RAW macrophages-conditioned medium as previously described (Liu et al., 2008; Li et al., 2018). Briefly, RAW264.7 cells were cultured in Dulbecco’s modified Eagle’s medium (DMEM), with 10% fetal bovine serum (FBS), 2 mM of l-glutamine, 100 units/ml penicillin, and 100 mg/ml streptomycin at 37°C in 5% CO_2_. All incubations were performed in cells under the three or four passages.

### Cell Viability Assays

RAW264.7 cell viability was assessed by the MTT-based spectrophotometric methods. Cells were seeded in 96-well microtiter plates at a density of 2.5 × 10^5^ cells/well. After 6 h, the culture medium was replaced with serum-free medium containing 0.05% DMSO (vehicle) or various concentrations of MH (25, 50, and 100 µM) for 24 h at 37°C and 5% CO_2_. Following treatment, cells were washed and incubated in 10% MTT, which was diluted in normal culture medium at 37°C until the visual color conversion occurred. Relative cytotoxicity was measured at 570-nm absorbance with FlexStation 3 multimode microplate reader (Molecular Device, Menlo Park, CA, USA). Cell viability was defined relative to vehicle-treated control, and the experiments were conducted three times independently.

For cell cultures, a stock of MH solution (10 mM) was prepared in DMSO and stored at −20°C. This stock solution was then further diluted in 0.05% DMSO before treatment of cells at the indicated concentrations; and these concentrations for cell treatment was based on previous *in vitro* bioactivity work (MadanKumar et al., 2014; Perumal et al., 2017).

### Cell Treatment Protocol

In experiments assessing the effects of MH on macrophage activation, LPS (100 ng/ml) was used to induce the inflammatory response in RAW264.7 cells as previously reported (Liu et al., 2008). Firstly, the timing of LPS for activating RAW264.7 cells was assessed. Then, cells were stimulated with LPS and cultured with treatment of MH (25, 50, and 100 µM) for 24 h at 37°C; parallel cultures were treated with an equivalent volume of vehicle for use as negative controls. Following treatment, TNF-α and IL-1β levels in the supernatants were quantified using commercial ELISA kits according to the manufacturer’s instructions. Each sample was tested in duplicate.

To further assess the effect of MH on TREM-1-mediated inflammatory response, RAW264.7 cells were exposed to LPS for 24 h in the presence or absence of MH (50 µM). Finally, cells were washed and collected by centrifugation for RNA harvesting and protein isolation for assessing the expression of TREM-1 and its downstream molecules. All measurements were performed in triplicate wells.

### Small Interfering RNA Transfection in RAW264.7 Cells

The TREM-1 small interfering RNA (siRNA) (siTREM-1) and non-targeted scrambled control siRNA (siNTC) were purchased from Santa Cruz Biotechnology (Delaware, CA, USA). Transient knockdown assays were performed using Lipofectamine 2000 (Invivogen, San Diego, CA, USA) according to the manufacturer’s instructions. Briefly, the cells were normally seeded into 6-well culture plates 24 h prior to transfection. Once the cells had reached 60–80% confluence, the cells were transfected using Lipofectamine 2000 and with 20 pmol/ml siRNAs, as follows: control group, siNTC group, and siTREM-1 group. Knockdown efficiency was determined by quantitative reverse transcriptase–polymerase chain reaction (RT-PCR) and western blotting. After RAW264.7 cells were transfected with siNTC or siTREM-1 for 48 h, cells were treated with 100 ng/ml LPS for 24 h in the presence of MH (50 µM) or vehicle; the control group was not transfected or treated with LPS. Afterwards, cells were collected to assess the corresponding indicators.

### Animals and Experimental Design

Pathogen-free male C57BL/6 mice (age, 6–8 weeks; body weight, 22∼25 g) were obtained from Shanghai Laboratory Animal Research Center (Shanghai, China). Mice were kept in an environmentally controlled room (23 ± 2°C, 55 ± 10% humidity) with a 12-h light-and-dark cycle and allowed free access to food and water. The study was performed in accordance with the Guiding Principles for the Care and Use of Laboratory Animals and approved by the Fudan University Animal Care Committee. Experimental design is outlined in **Figure 1B**. MH was dissolved in DMSO, and mice were orally given 50 mg/kg of MH once a day for five consecutive days before challenge with CCl_4_. The dosage of MH was chosen according to previous studies in mice (Lee et al., 2016; Naowaboot et al., 2016). For the induction of acute liver injury, a single dose of 1 ml/kg CCl_4_ (1:1 v/v in olive oil) was injected intraperitoneally (i.p.) in C57BL/6 mice. Mice were randomly divided into four groups, each consisting of eight animals. The groups were as follows: Group I was the normal control in which mice were not treated with either CCl_4_ or MH but were given an equal volume olive oil (i.p.) and orally administered with an equal volume of DMSO; Group II was MH-treated control, and mice were given MH orally and injected with olive oil; Group III mice were injected with CCl_4_ and administered with an equal volume of DMSO for 5 days; and Group IV was MH-pretreated group in which mice were given morin for 5 days before challenge with CCl_4_. All mice were sacrificed at 24 h after CCl_4_ injection, blood was collected, and liver samples were harvested upon euthanasia.

### Assay for Serum AST and ALT Levels

Levels of serum aspartate transaminase (AST) and alanine transaminase (ALT) were determined with a commercial assay kit according to the manufacturer’s recommendations (Nanjing Jiancheng Biological Technology, Inc., Nanjing, China). Liver enzyme activities were shown in international unit per liter (U/L).

### Estimation of Oxidative Stress and Antioxidant Property in the Liver

The liver of each mice was isolated, washed, and perfused with chilled normal saline. Approximately 1 g was minced and homogenized in ice-cold phosphate buffer (140 mM of KCl, 20 nM of phosphate, and pH 7.4). The homogenate was centrifuged at 1,500 *g* for 15 min at 4°C. The supernatant was used for the estimation of lipid peroxidation and oxidative stress markers.

MDA formation in mouse livers by lipid peroxidation MDA assay kit according to the protocol provided by the manufacturer. MDA levels were calculated using the standard curve of MDA, and its level is expressed in nmol/mg of protein liver tissue. Total glutathione (GSH plus oxidized glutathione (GSSG)) and GSH were determined using total glutathione assay kit, and total SOD activity was estimated using total SOD assay kit with NBT according to the protocol provided by the manufacturer. GSH and GSSG activities were expressed as nmol/mg of protein, and SOD activity was expressed as U/mg of protein.

### Analysis of Liver Histopathology

For histological analysis, liver tissue specimens were fixed in 10% buffered formalin and embedded in paraffin, cut at thicknesses of 4 µm, and then stained with hematoxylin and eosin (H&E) according to standard procedure. Necrosis in the liver sections was assessed by a registered pathologist in a blinded manner and graded by a semiquantitative score from 0 to 3 as described previously (Horrillo et al., 2007).

### Immunohistochemistry and Analysis of Histological Markers

The liver tissue sections were dewaxed, hydrated, and pretreated with the heat-induced antigen retrieval technique. Sections were blocked and then incubated with the respective primary antibody as described previously (Li et al., 2018). Primary antibodies were incubated on the sections overnight at 4°C at the following concentrations: TREM-1 antibody, 1:100; TLR4 antibody, 1:50; and F4/80 antibody, 1:50. All antibodies were diluted in Tris-buffered saline (TBS)–2% bovine serum albumin. Negative-control antibodies consisted of species-matched and, where appropriate, Ig G (IgG) subclass-matched Ig fractions, used at the same dilution as the secondary antibodies. Color development was induced using 3,3′-diaminobenzidine (DAB) tetrachloride during an incubation period of 5 to 10 min. With the use of this substrate, specific staining was visualized by light microscopy.

For quantification of the numbers of liver macrophages and TREM-1-positive cells in sections, six non-overlapping randomly selected fields of view per slide at ×400 magnifications were examined; and five mice of each group were examined. The result was expressed as the number of F4/80^+^ cells or TREM-1^+^ cells per high-power field (HPF) (Li et al., 2018).

### Detection of Cell Death by TUNEL Staining

TUNEL (terminal deoxynucleotidyl transferase mediated dUTP nick-end labeling) staining was performed to assess death hepatocytes in liver sections. Formalin-fixed, paraffin-embedded tissue sections were stained by using a One-step TUNEL Apoptosis Detection Kit (Alexa Fluor 647, YEASEN, Shanghai, China) according to the manufacturer’s instructions. Finally, slides were counterstained with DAPI and hematoxylin solution. The number of TUNEL-positive cells (red) was counted on five fields of ×400 magnification per slide, and five mice of each group were examined. The results were expressed as the mean number of TUNEL-positive cells.

### Quantitative Real-Time PCR Analysis

RNA was extracted from frozen liver tissues and RAW264.7 cells using TRIzol reagent following the manufacturer’s protocol. RNA was extracted and reverse transcribed with random hexamers and avian myeloblastosis virus reverse transcriptase using a commercial kit (Perfect Real Time, SYBR^®^ PrimeScriP^TM^, TaKaRa, Japan). Quantitative RT-PCR was performed for assessment of gene expression using ABI Prism 7500 Sequence Detection system (Applied Biosystems, Tokyo, Japan). The relative changes were normalized to GAPDH mRNA using the formula 2^−ΔΔCt^, where ΔΔCt represents ΔCt values normalized with the mean ΔCt of control samples as described previously (Li et al., 2016a; Li et al., 2018). Sequences for target genes were purchased from Sangon Biotech Co., Ltd. (Shanghai, China) and are listed in **Table 1**.

**Table 1:** Mouse primer sequences used for quantitative real-time PCR.

Target gene	Forward primers (5′–3′)	Reverse primers (5′–3′)
**TLR4**	CCTGAGCAAACAGCAGAGGA	CCATGTGTTCCATGGGCTCT
**HO-1**	AGCCCCACCAAGTTCAAACA	TCTCTGCAGGGGCAGTATCT
**Nrf2**	TCTCCTAGTTCTCCGCTGCT	TGGTGTCTGTCTGGATGTGC
**TNF-α**	GGACTAGCCAGGAGGGAGAA	CGCGGATCATGCTTTCTGTG
**IL-1β**	ACCTAGCTGTCAACGTGTGG	TCAAAGCAATGTGCTGGTGC
**TREM-1**	TCCTATTACAAGGCTGACAGAGCGTC	AAGACCAGGAGAGGAAACAACCGC
**IL-6**	GGAGTCACAGAAGGAGTGGC	CGCACTAGGTTTGCCGAGTA
**GAPDH**	CTCGTGGTTCACACCCATCA	CTCGTGGTTCACACCCATCA

PCR, polymerase chain reaction; TLR4, toll-like receptor 4; HO-1, heme oxygenase-1; Nrf2, nuclear factor erythroid 2-related factor 2; TNF-*α*, tumor necrosis factor *α*; IL, interleukin; TREM-1, triggering receptor expressed on myeloid cells-1.

### Isolation of Cytoplasmic and Nuclear Proteins

To obtain cytoplasmic and nuclear fractions from whole liver tissue or cultured cells, nuclear and cytoplasmic proteins were prepared as described before (Li et al., 2016a; Li et al., 2018). In brief, nuclear and cytosolic proteins were extracted using NE-PER^®^ (Pierce Biotechnology, Rockford, IL, USA) according to manufacturer’s instructions. Protein concentrations were measured using Bicinchoninic Acid Protein Colorimetric Assay kits (BMI, Shanghai, China) with bovine serum albumin as standard.

### Western Blot Analysis

Western blots were used to assess levels of expression of TREM-1, TLR4, inhibitor of kappa B α (IκBα), NF-κB p65, nuclear factor erythroid 2-related factor 2 (Nrf2), and heme oxygenase-1 (HO-1) subunit proteins. Samples (30 μg of protein/lane) were separated by sodium dodecyl sulfate–polyacrylamide electrophoresis (SDS-PAGE) and transferred to polyvinylidene difluoride (PVDF) membranes. After blockade of nonspecific binding sites with 5% (w/v) non-fat milk power for 1 h, membranes were washed with TBST and subsequently incubated in Tween phosphate-buffered saline at 4°C for 24 h with the following primary antibodies: TREM-1 antibody (1:1,000 dilution), TLR4 antibody (1:1,500), IκBα antibody (1:2,000), NF-κB/p65 antibody (1:2,500), Nrf2 antibody (1:2,000), and HO-1 antibody (1:1,500). Thereafter, the membranes were washed by TBS/Tween followed by incubation with corresponding secondary antibody for 2 h at room temperature. Protein bands were visualized using ECL substrate (Bio-Rad Laboratories) according to the instructions of the manufacturer. Membranes were also incubated with antibodies against GAPDH (1:5,000 dilution) and Lamin B1 (1:3,000), as internal controls for cytosolic and nuclear proteins, respectively. Densities were quantified using Image J software (NIH, Bethesda, Maryland, USA).

### Statistical Analysis

All data are expressed as the mean ± standard error of mean (SEM), unless otherwise stated. Statistical analyses were performed using GraphPad Prism 8 statistical software (La Jolla, CA, USA). Comparisons among three or more groups were performed by one-way analysis of variance (ANOVA) with post-hoc Tukey’s multiple comparison tests or by two-tailed unpaired Student’s *t*-tests. Comparisons between two independent groups were performed using a two-sample *t*-test. For all analyses, *P*-values of less than 0.05 were considered to be statistical significance.

## Results

### Effect of MH on CCl_4_-Induced Liver Injury in Mice

The protective effect of MH against acute liver injury was confirmed by analysis of serum aminotransferase levels and histopathological findings by H&E staining. As shown in **Figure 1C**, compared with those in DMSO/oil or MH/oil group mice, the levels of serum ALT and AST significantly increased in response to CCl_4_ injection in mice; however, CCl_4_-injected mice with MH (50 mg/kg) pretreatment remarkably inhibited the levels of the two transaminases than in those mice with vehicle pretreatment. Notably, pretreatment of mice with MH alone for 5 days did not show any significant influence in liver enzymes than did the DMSO/oil group mice, which signified a nontoxic effect of MH at this dosage on the liver from mice. Furthermore, serum aminotransferase levels paralleled the histopathological alterations. As shown in **Figure 1D**, liver sections from the oil-treated control mice receiving DMSO or MH showed normal cellular architecture and did not display any hepatocyte necrosis with distinct hepatic cells and sinusoidal spaces. In contrast, the CCl_4_-exposed groups exhibited obvious destruction of hepatic lobule structure, hyperemia, inflammatory infiltration, and large areas of necrosis within the liver lobules. However, pretreatment with MH of CCl_4_-injected mice dramatically alleviated liver inflammation and hepatocyte necrosis than did vehicle pretreated to those mice (**Figure 1D**). These findings were validated by the scores for necrosis in livers (**Figure 1E**).

To further confirm that MH suppressed hepatic inflammation in acute liver injury in mice, we analyzed the macrophage infiltration in injured liver by immunostaining with an antibody against F4/80. Our results demonstrated that F4/80-positive macrophages were detected in sinusoids of both the DMSO/oil group and MH/oil group. Notably, a massive accumulation of F4/80-positive macrophages could be observed in injured livers from mice induced by CCl_4_. However, this increase in macrophages recruitment in livers was remarkably reduced by pretreatment with MH (**Figure 1F**). Consistent with this observation, the number of F4/80 staining cells per HPF obviously increased in the liver of mice induced by CCl_4_, and this increase was significantly lower in MH-treated injured mice than in vehicle-treated mice (29.23 ± 1.05/HPF vs. 44.97 ± 0.76/HPF, *P* < 0.001; **Figure 1G**).

Moreover, we used TUNEL assay to assess the effect of MH on the hepatocyte death in the liver after challenge CCl_4_. As shown in **Figures 1H, I**, CCl_4_ injection markedly increased the number of hepatocytes apoptosis than in the oil-treated groups. However, the number of TUNEL-positive cells in MH/CCl_4_ group was significantly decreased as compared with that in the DMSO/CCl_4_ group.

On the whole, our results demonstrated that pretreatment with MH mitigated CCl_4_-induced liver injury, inflammation, and cell death in mice.

### Effect of MH on TREM-1 Expression in Livers From Mice Induced With CCl_4_

**Figure 2:**
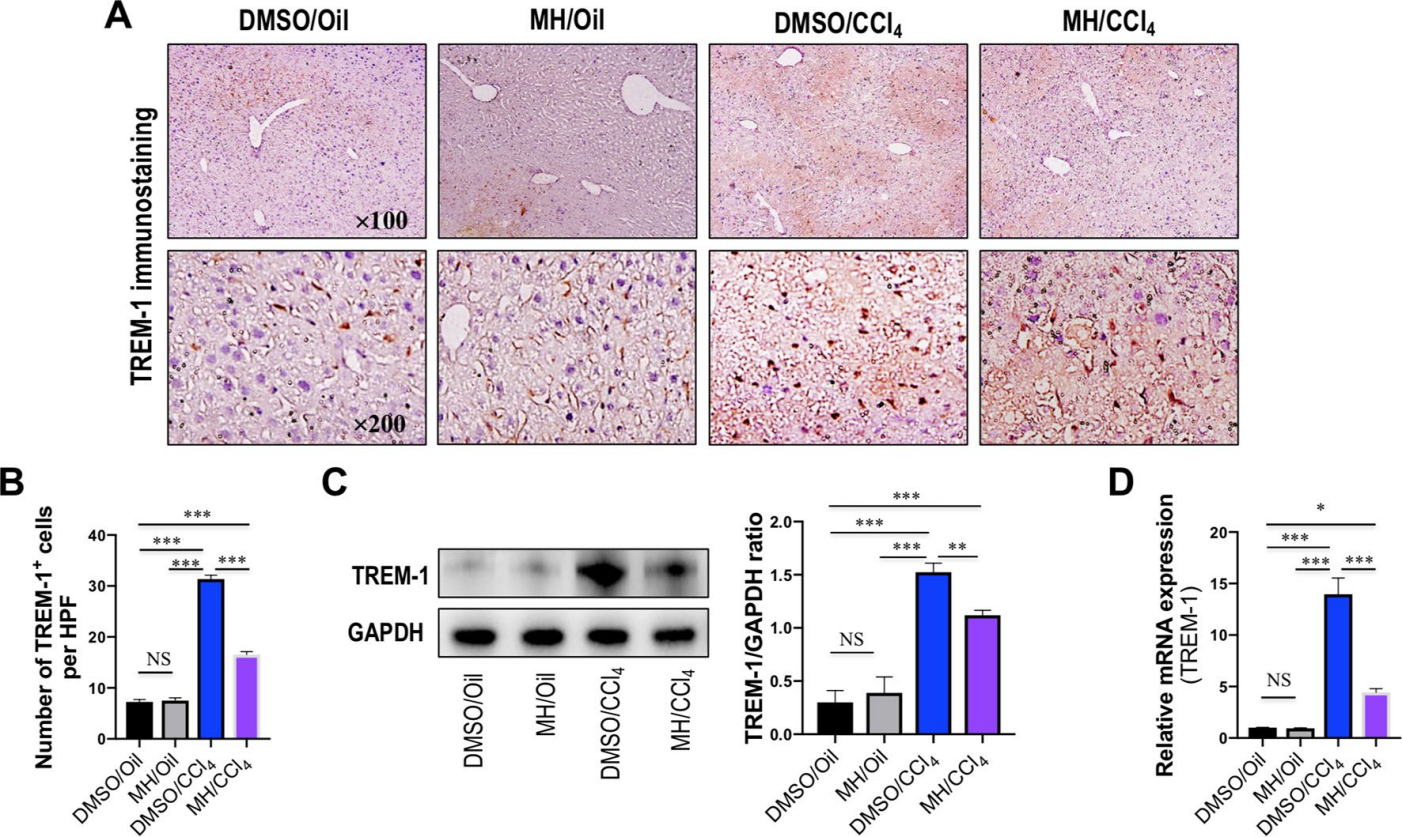
Effect of morin hydrate (MH) on triggering receptor expressed on myeloid cells-1 (TREM-1) expression in livers from mice induced by CCl_4_. **(A)** Expression of TREM-1 in the liver was determined by immunohistochemistry. Original magnification, ×100 (upper column) and ×200 (lower column). **(B)** Quantification of TREM-1-positive cells per high-power field (HPF) in liver sections from each group. Results mean of six fields and *n* = 5 for each group. **(C)** Western blot analysis of TREM-1 protein in liver lysates from each group; results normalized relative to expression of GAPDH. **(D)** Hepatic TREM-1 mRNA expression was measured by quantitative reverse transcriptase–polymerase chain reaction (RT-PCR). Results are shown as fold change compared with dimethyl sulfoxide (DMSO/oil group and GAPDH serving as loading control (*n* = 5). *P < 0.05, **P < 0.01, ***P < 0.001. NS indicates not significant.

Expression and localization of TREM-1 in livers of mice from each group were determined by immunohistochemistry. As shown in **Figure 2A**, immunostaining of livers from oil-treated control mice revealed that TREM-1 has weak signals and was localized predominantly in the sinusoids. In contrast, 24 h after injection of CCl_4_, greater TREM-1 immunoreactivity was observed in the periportal areas and necrotic areas of the liver. However, pretreatment with MH of CCl_4_-injected mice remarkably decreased the immunoreactivity of TREM-1 in the sinusoids than did pretreatment with DMSO in mice (**Figure 2A**). Consistent with this finding, CCl_4_ injection obviously increased the number of TREM-1 staining cells per HPF, and the increase was significantly lower in MH-treated injured mice than in DMSO-treated mice (**Figure 2B**). Furthermore, our western blotting revealed that pretreatment with 50 mg/kg MH of CCl_4_-injected mice significantly reduced the levels of TREM-1 protein expression than did pretreatment with DMSO of those animals (**Figure 2C**). In addition, intrahepatic expression of TREM-1 mRNA was also elevated at 24 h after CCl_4_ injection, but this gene was reduced remarkably in MH-pretreated than in DMSO-pretreated mice induced by CCl_4_ (**Figure 2D**).

Taken together, our data demonstrated that MH inhibited TREM-1 expression in injured livers of mice induced by CCl_4_.

### Effect of MH on TLR4 Expression and Localization in the Liver of CCl_4_-Treated Mice

**Figure 3:**
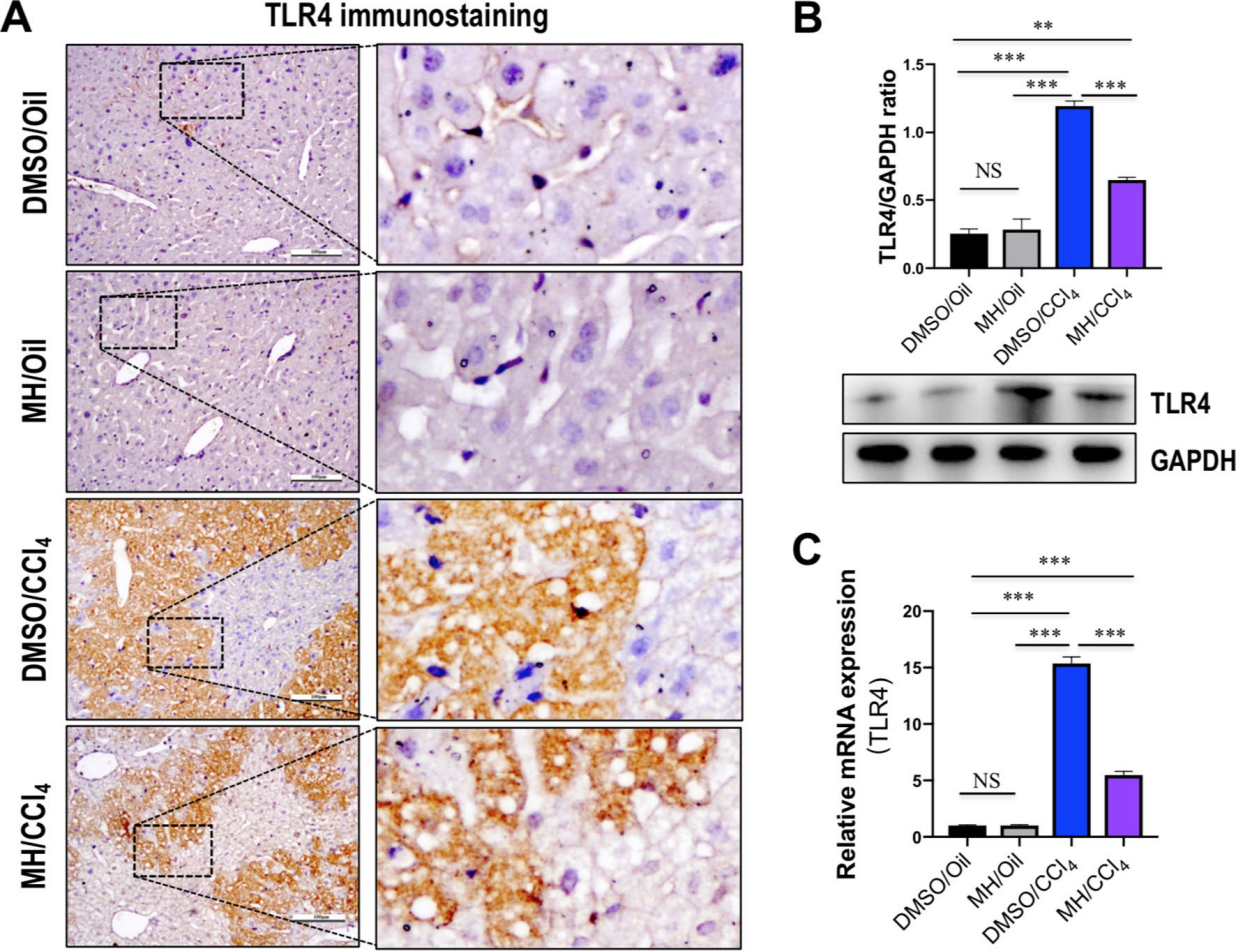
Effect of morin hydrate (MH) on toll-like receptor 4 (TLR4) expression and localization in the liver of CCl_4_-treated mice. **(A)** Expression and distribution of TLR4 in the liver were determined by immunohistochemistry. Original magnification, ×200 (left column) and ×400 (right column). Boxed regions in the left column were enlarged. **(B)** Western blot analysis of TLR4 protein of liver tissue samples; results normalized relative to expression of GAPDH. **(C)** Hepatic TLR4 mRNA expression was measured by quantitative reverse transcriptase–polymerase chain reaction (RT-PCR). Results are shown as fold change compared with dimethyl sulfoxide (DMSO)/oil group and GAPDH served as loading control (*n* = 5). **P < 0.01, ***P < 0.001. NS indicates not significant.

Because TREM-1 is reported to amplify the inflammatory response initiated by TLR engagement (Ornatowska et al., 2007; Tammaro et al., 2017), we further investigated whether TLR4 signaling was inhibited by MH in acute liver injury. As shown in **Figure 3A**, immunohistochemistry showed that the liver sections from oil-treated control mice showed weak constitutive expression of TLR4 on vascular and sinusoidal endothelial cells and no expression on hepatocytes, similar to our previous studies (Li et al., 2016a; Li et al., 2016b). TLR4 expression signaling was increased on vascular, sinusoidal endothelial cells and hepatocytes after CCl_4_ injection, but overall TLR4 immunoreactivity was reduced by MH pretreatment when compared with DMSO-pretreated control (**Figure 3A**). Consistent with this result, pretreatment with MH of CCl_4_-injected mice significantly reduced the levels of TLR4 protein expression than in vehicle-pretreated animals (**Figure 3B**). In addition, intrahepatic expression of TLR4 mRNA was also induced at 24 h after CCl_4_ injection, but this gene was reduced remarkably in MH-pretreated than in DMSO-pretreated mice induced by CCl_4_ (**Figure 3C**).

### Effect of MH on NF-κB Nuclear Translocation and Proinflammatory Cytokines Expression in the Liver of CCl_4_-Treated Mice

**Figure 4:**
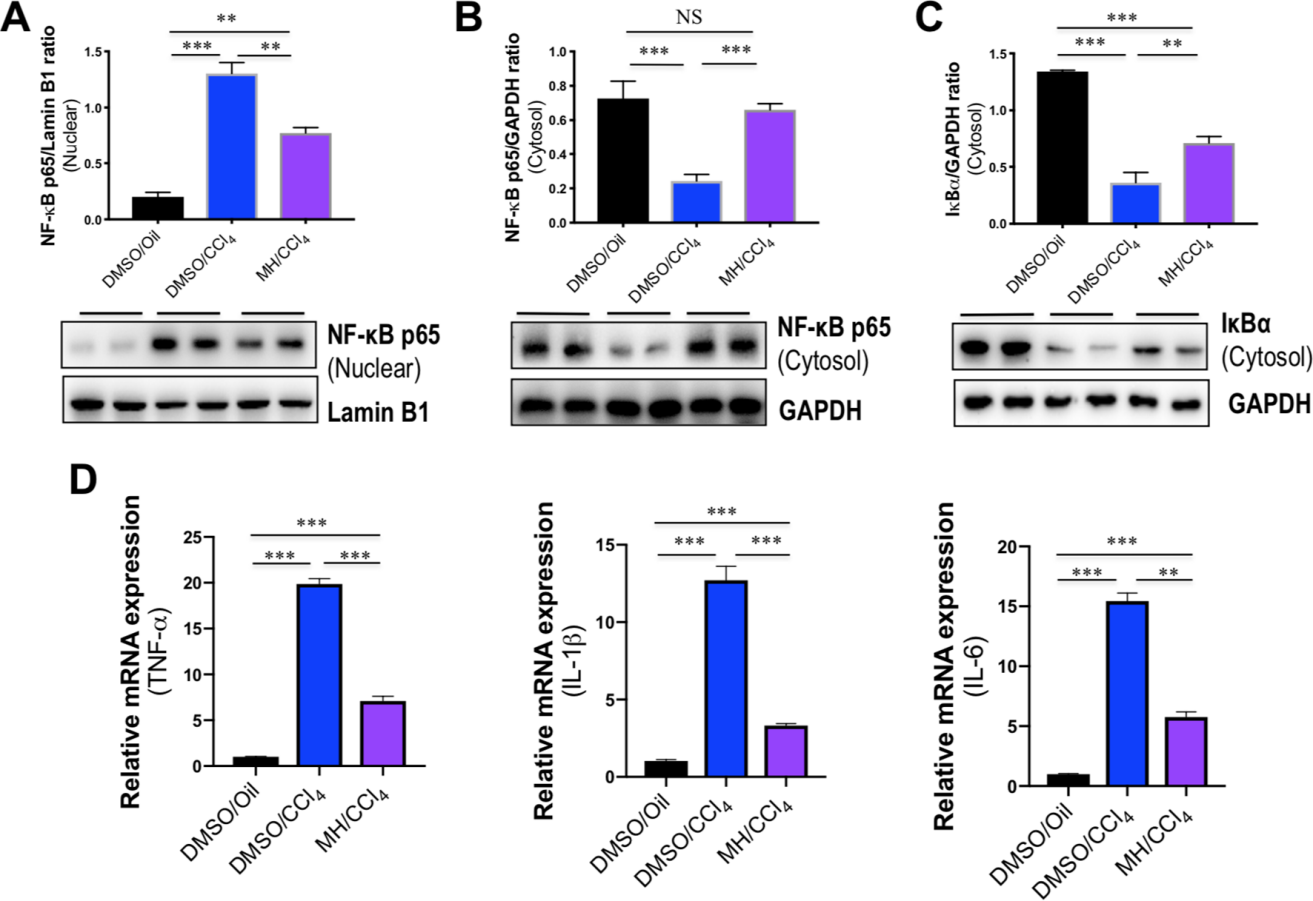
Effect of morin hydrate (MH) on CCl_4_-induced nuclear factor-kappa B (NF-κB) translocation and proinflammatory cytokine expression in the liver. **(A)** Western blot analysis of NF-κB p65 in the nuclear fractions of liver tissue; results normalized relative to expression of Lamin B1. **(B)** Western blot analysis of NF-κB p65 protein in the cytosolic fractions of liver tissue; results normalized relative to expression of GAPDH. **(C)** Western blot analysis of I-κBα protein in the cytosolic fractions of liver tissue samples; results normalized relative to expression of GAPDH. **(D)** Hepatic proinflammatory cytokine gene of tumor necrosis factor α (TNF-α), interleukin (IL)-1β, and IL-6 was determined by quantitative reverse transcriptase–polymerase chain reaction (RT-PCR), and results are shown as fold change compared with dimethyl sulfoxide (DMSO)/oil group and GAPDH served as loading control (*n* = 5). **P < 0.01, ***P < 0.001. NS indicates not significant.

It is well established that NF-κB is activated by the TLR signaling pathway. Thus, we examined the effect of MH on the activation of NF-κB in livers, and western blot analysis of the nuclear translocation of the NF-κB p65 subunit was performed in liver tissues. Our results revealed that the level of nuclear NF-κB p65 in the liver was increased by CCl_4_ injection and that this change was inhibited by MH administration (**Figure 4A**). In contrast, CCl_4_ injection led to reduction in the level of cytosolic NF-κB p65 in the liver, but this decrease was restored by MH administered to CCl_4_-injected mice (**Figure 4B**). Additionally, we also found that the intrahepatic level of IκBα in the cytosol was lower in the CCl_4_-treated than in DMSO/oil group, and this reduction was remarkably inhibited by MH pretreatment in CCl_4_-induced mice (**Figure 4C**). As the cytosolic IκBα was associated with NF-κB translocation from cytosol to nuclear, this result confirmed that MH inhibited nuclear translocation of NF-κB p65 with reduced intrahepatic IκBα degradation.

The transcription factor NF-κB plays an important role in the inflammatory response as NF-κB activation can activate the transcription of various proinflammatory genes and regulate inflammation (Seki et al., 2007). Therefore, the expression of hepatic proinflammatory cytokines such as TNF-α, IL-1β, and IL-6 mRNA was also assayed by quantitative RT-PCR. As shown in **Figure 4D**, the levels of TNF-α, IL-1β, and IL-6 mRNA in livers increased significantly in response to CCl_4_ challenge, while MH pretreatment remarkably suppressed the increase of those genes in the liver.

Taken together, these results indicated that pretreatment with MH markedly mitigated CCl_4_-induced liver injury through inhibition of TLR4/NF-κB-mediated inflammatory response.

### Effect of MH on the Production of Proinflammatory Cytokines in LPS-Stimulated RAW264.7 Cells

**Figure 5:**
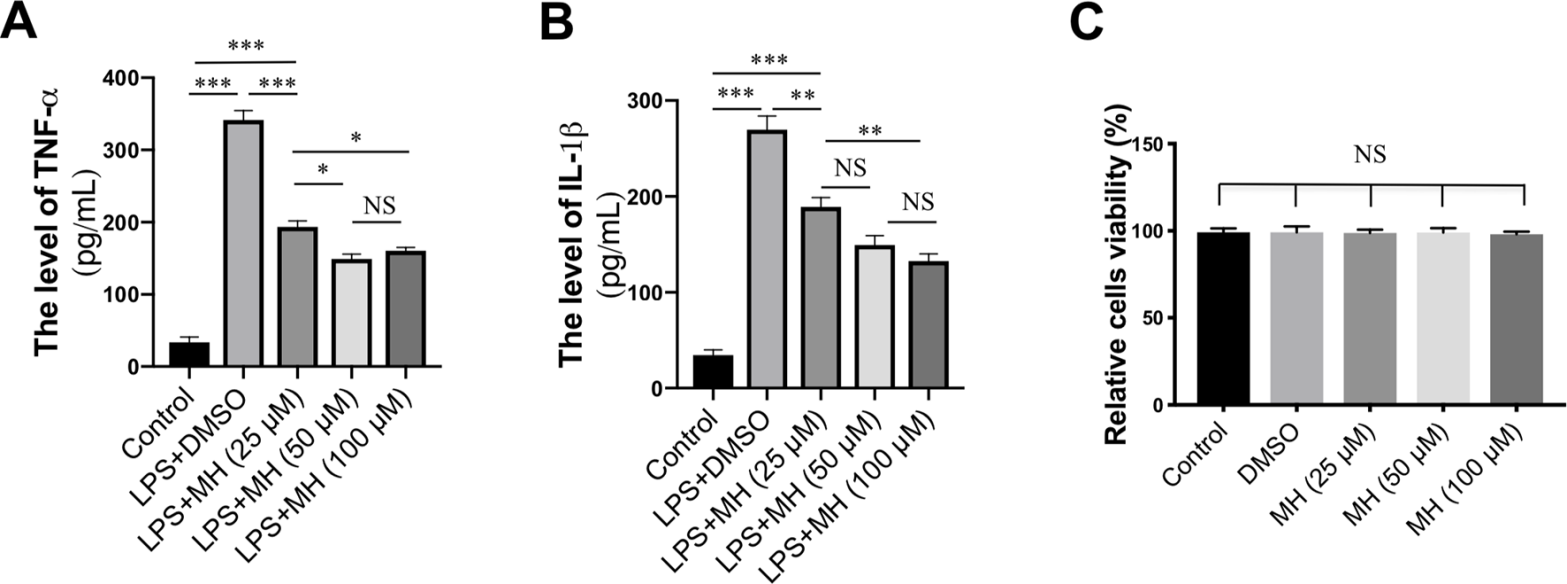
Effect of morin hydrate (MH) on the production of proinflammatory cytokines in lipopolysaccharide (LPS)-stimulated RAW264.7 cells. RAW264.7 cells were treated with 25, 50, and 100 µM of MH and co-cultured with or without LPS. **(A**–**B)** The level of TNF-α and IL-1β in the supernatants was measured by enzyme-linked immunosorbent assay (ELISA). Data are expressed as mean ± SEM from three independent experiments. **(C)** RAW264.7 cells were treated with various concentrations of MH (25, 50, and 100 µM) for 24 h, and cell viability was assessed by MTT assay. *P < 0.05, **P < 0.01, ***P < 0.001. NS indicates not significant.

To study the mechanisms underlying the anti-inflammatory effects mediated by MH, RAW264.7 murine macrophage cells were treated with LPS and various concentrations of MH. As expected, LPS-activated RAW264.7 cells resulted in a significant increase in the production of TNF-α and IL-1β cytokines. However, those mediators of the inflammatory response were remarkably inhibited by MH in a dose-dependent manner (**Figures 5A, B**). In addition, to avoid any cytotoxic effects of MH, the cytotoxicity of MH was assessed; and our result demonstrated that cell viability was unaffected by those MH concentrations as confirmed by MTT assay (**Figure 5C**). Therefore, the concentration of MH at 50 µM was used for the following study.

### Effect of MH on the TREM-1/TLR4 Signaling Molecules From LPS-Stimulated RAW264.7 Cells

**Figure 6:**
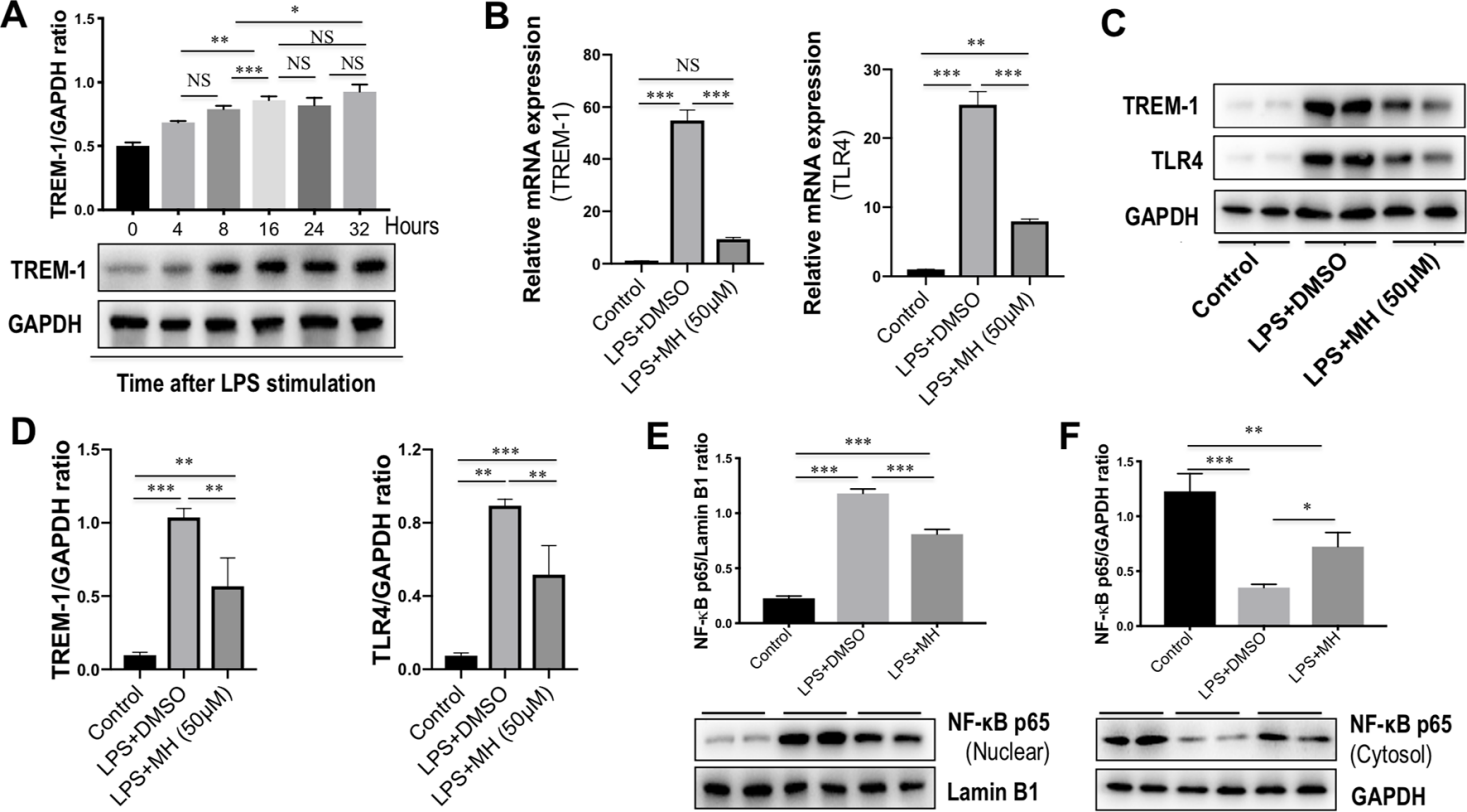
Effect of morin hydrate (MH) on the triggering receptor expressed on myeloid cells-1 (TREM-1)/toll-like receptor 4 (TLR4) signaling molecules in lipopolysaccharide (LPS)-stimulated macrophages. **(A)** RAW264.7 cells were stimulated with LPS (100 ng/ml) and collected at the indicated time points. TREM-1 expression was assessed using western blot; results normalized relative to expression of GAPDH. **(B)** RAW264.7 cells were stimulated with LPS (100 ng/ml) and treated with MH (50 µM) or vehicle (DMSO) for 24 h; and quantification reverse transcriptase–polymerase chain reaction (RT-PCR) for TREM-1 and TLR4 mRNA expression. **(C**, **D)** Western blot analysis of TREM-1 and TLR4 protein levels; results normalized relative to expression of GAPDH. Blot shown is representative of three experiments with similar results. **(E)** Western blot analysis of NF-κB p65 in the nuclear fractions of RAW264.7 cells; results normalized relative to expression of Lamin B1. **(F)** Western blot analysis of NF-κB p65 protein in the cytosolic fractions of RAW264.7 cells; results normalized relative to expression of GAPDH. *P < 0.05, **P < 0.01, ***P < 0.001. NS indicates not significant.

To identify the effects of TREM-1 on inflammatory response, RAW264.7 cells were stimulated with LPS for different time points. As shown in **Figure 6A**, our result revealed that the level of TREM-1 protein was gradually upregulated in RAW264.7 cells in response to LPS in a time-dependent manner. To investigate the effect of MH on TREM-1 expression in LPS-stimulated macrophages, RAW264.7 macrophages were stimulated *in vitro* with MH (50 µM) and LPS (100 ng/ml) or DMSO for 24 h. The result in this study revealed that the expression of TREM-1 gene was increased obviously in cells after stimulation with LPS than in the cells with vehicle. However, MH treatment significantly blocked LPS-induced TREM-1 gene expression on RAW264.7 cells (**Figure 6B**). Consistent with this result, our western blot analysis also demonstrated that LPS-treated macrophages in the presence of MH resulted in the decrease of TREM-1 protein levels than in DMSO-treated cells (**Figures 6C, D**). Similarly, as shown in **Figures 6B–D**, MH also remarkably downregulated the levels of the TLR4 gene and protein expression in LPS-stimulated RAW264.7 cells *in vitro*. Furthermore, our results also demonstrated that LPS induced a remarkably increase of nuclear translocation of NF-κB p65 subunit in RAW264.7 cells. However, application of MH potently inhibited this translocation *in vitro* (**Figures 6E, F**).

### Effect of MH on the TREM-1/TLR4 Signaling Molecules From LPS-Stimulated RAW264.7 Cells After TREM-1 Knockdown

**Figure 7:**
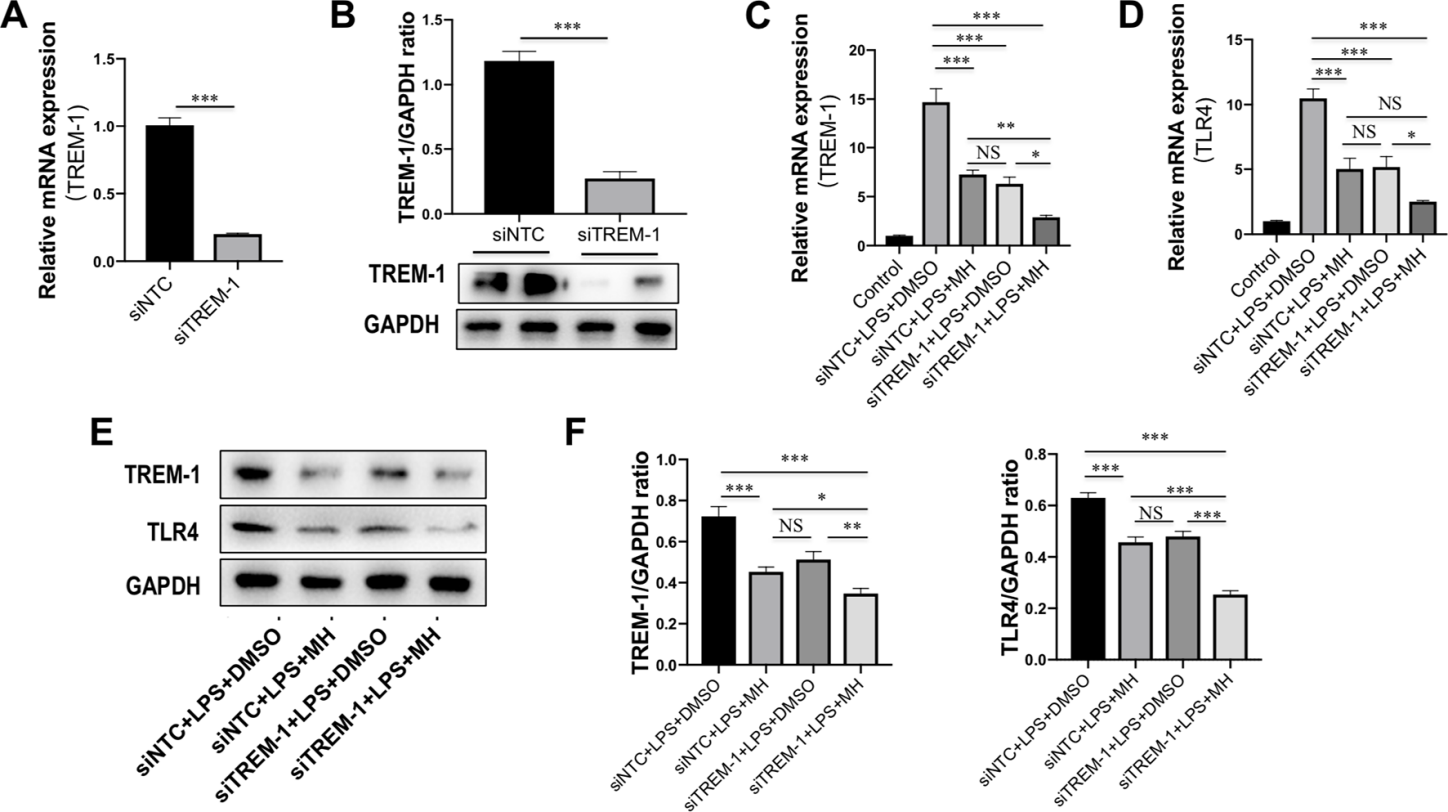
Effect of morin hydrate (MH) on expression of the triggering receptor expressed on myeloid cells-1 (TREM-1) and toll-like receptor 4 (TLR4) in (LPS)-stimulated macrophages after TREM-1 knockdown. **(A**, **B)** RAW264.7 cells that had been transfected with siNTC or siTREM-1 for 24 h, and then cells were treated for 24 h with 100 ng/mL LPS. The transfected effective was confirmed by assessing the gene **(A)** and protein expression **(B)**. TREM-1 mRNA was assayed by quantitative reverse transcriptase–polymerase chain reaction (RT-PCR), and TREM-1 protein expression was examined by Western blot. **(C**, **D)** The levels of TREM-1 and TLR4 mRNA expression were determined by quantitative RT-PCR, and results are shown as fold change compared with control group and GAPDH served as loading control (*n* = 5). RAW264.7 cells had been transfected with siNTC or siTREM-1 and then treated for 24 h with 100 μg/mL LPS at the presence of MH (50 μM) or DMSO. **(E**, **F)** Western blot for TREM-1 and TLR4 protein expression. Representative blots are shown together with the corresponding densitometric quantification values, which were normalized based on GAPDH expression. RAW264.7 cells had been transfected with siNTC or siTREM-1 and then treated for 24 h with 100 μg/mL LPS at the presence of MH (50 μM) or DMSO. *P < 0.05, **P < 0.01, ***P < 0.001. NS indicates not significant.

To further determine the mechanisms by which MH suppressed the inflammatory response in macrophages through inhibition of TREM-1 signaling pathway, RAW264.7 cells were transfected with TREM-1-specific siRNA, and the knockdown of TREM-1 was confirmed by RT-PCR and western blot (**Figures 7A, B**). Then, the MH group and model group were stimulated by LPS (100 ng/mL) for 24 h with the presence of MH (50 μM) or DMSO. Our result demonstrated that there was no difference between the siNTC + LPS + MH group and the siTREM-1 + LPS + DMSO group in expression of the TREM-1 and TLR4 mRNA (**Figures 7C, D**) and protein (**Figures 7E, F**). The mRNA and protein levels of TREM-1 and TLR4 in the siNTC + LPS + DMSO group were remarkably increased than in the siNTC + LPS + MH group. Of note, compared with the siTREM-1 + LPS + DMSO group, the levels of TREM-1 and TLR4 in the siTREM-1 + LPS + MH group were further decreased (**Figures 7C–E**). Taken together, these results indicated that MH could suppress TREM-1 and TLR4 expression in LPS-stimulated RAW264.7 cells, and this inhibitory effect had enhanced by TREM-1 knockdown.

### Effect of MH on Oxidative Stress in the Liver of CCl_4_-Treated Mice

**Figure 8:**
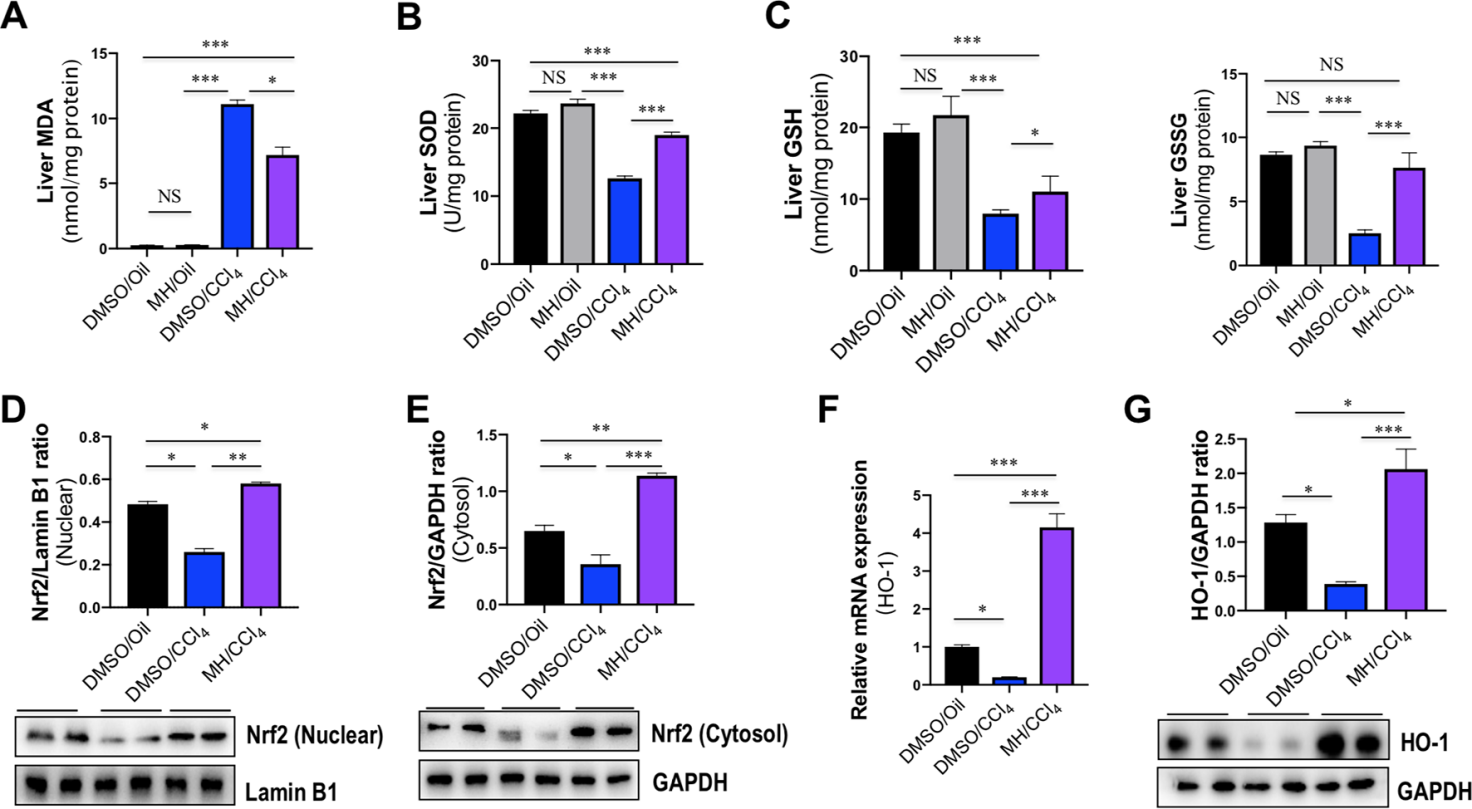
Effect of morin hydrate (MH) on oxidative stress and nuclear factor erythroid 2-related factor 2 (Nrf2)/heme oxygenase-1 (HO-1) pathway in the liver of CCl_4_-treated mice. **(A)** Lipid peroxidation was assessed in terms of malondialdehyde (MDA) formation (*n* = 6 for each group). **(B)** Oxidative stress was assessed in terms of Cu/Zn SOD activity. Values are expressed as mean ± SEM (*n* = 6 for each group). **(C)** Oxidative stress was assessed in terms of glutathione (GSH) and oxidized glutathione (GSSG). *n* = 6 for each group. **(D)** Western blot analysis of Nrf2 protein in nuclear fractions of liver tissue; results normalized relative to expression of Lamin B1. **(E)** Western blot analysis of Nrf2 protein expression in cytosolic fractions of liver tissue; results normalized relative to expression of GAPDH. **(F)** Hepatic HO-1 mRNA expression was measured by quantitative reverse transcriptase–polymerase chain reaction (RT-PCR). Results are shown as fold change compared with DMSO/oil group and GAPDH served as loading control (*n* = 5 for each group). **(G)** Western blot analysis of HO-1 protein expression in the lysed liver tissue; results normalized relative to expression of GAPDH (*n* = 3). *P < 0.05, **P < 0.01, ***P < 0.001. NS indicates not significant.

To determine the oxidative stress in our study, we evaluated the MDA formation in the liver as evidence of lipid peroxidation. The administration of CCl_4_ remarkedly increased hepatic MDA content, whether animals were pretreated with vehicle or MH (11.11 and 7.16 nmol/mg protein, respectively), than in the control mice (0.25 nmol/mg protein). However, MH pretreatment led to a significant amelioration in CCl_4_-induced increase in hepatic MDA content (**Figure 8A**).

Liver GSH levels and SOD activity were measured as markers of oxidative stress at the hydrophilic level. Compared with normal control, administration of CCl_4_ significantly decreased the activity of SOD in the liver; however, those reductions were markedly restored by MH pretreatment in the liver of CCl_4_-treated mice when compared with vehicle-pretreated animals (12.61 vs. 18.98 U/mg protein) (**Figure 8B**). Similarly, CCl_4_-induced oxidative stress led to a significant decrease in hepatic GSH and GSSG in mice, while MH administration prevented the decrement in GSH induced by CCl_4_. Moreover, in the group receiving CCl_4_and MH, GSSG increased significantly (**Figure 8C**).

### Effect of MH on Nrf2/HO-1 Pathway in the Liver of CCl_4_-Treated Mice

Nrf2 is a cellular sensor of oxidative stress, and Nrf2 is crucial for antioxidant response element (ARE)-mediated induction of detoxifying enzymes, anti-oxidative stress genes, and other target genes involved in cellular protection (Bataille and Manautou, 2012). To further determine the molecular mechanism of oxidative stress induced by MH in CCl_4_-induced liver injury, we firstly examined Nrf2 expression and translocation by western blotting; the result revealed that the level of nuclear Nrf2 expression was decreased in CCl_4_-exposed mice compared with the control mice but was remarkably increased in those mice pretreated with MH (**Figure 8D**). Similarly, the level of cytoplasmic Nrf2 expression was also decreased in the mice receiving CCl_4_ alone compared with the control mice. In contrast, the mice pretreatment with MH showed a higher level of cytoplasmic Nrf2 with respect to that in the mice of vehicle-treated control (**Figure 8E**). Simultaneously, we also assessed the Nrf2’s downstream target HO-1 mRNA and protein expression by RT-PCR and western blot, respectively. As shown in **Figures 8F, G**, CCl_4_ administration led to decrease in the levels of HO-1 gene and protein expression in the injured liver as compared with normal control. However, pretreatment with MH (50 mg/kg) of CCl_4_-injected mice led to a substantial induction of gene and protein expression of HO-1 in livers. Altogether, these results suggested that MH pretreatment could effectively upregulate the Nrf2 and HO-1 expression as well as promote nuclear translocation of Nrf2.

## Discussion

The present study provided further evidence to support that MH could protect against acute CCl_4_-induced liver damage by inhibiting TREM-1-mediated inflammatory response and attenuating oxidative stress in the liver. Most importantly, our finding strongly suggested that TREM-1/TLR4/NF-κB signaling pathway might play a vital role in the pathogenesis of acute liver injury, and its inhibition by MH could exert beneficial effects in the prevention of acute hepatic damage.

A growing body of evidence indicates that natural products and their derivatives could prevent the deleterious effects of toxic agents by scavenging free radicals or modulating inflammatory response (Conner and Grisham, 1996; Lu et al., 2012; Singh et al., 2015; Hassan et al., 2017). Notably, MH, one of the most widely distributed flavonoids in plants, possesses strong free radical scavenging ability and potent hepatoprotective effects (Caselli et al., 2016; Lee et al., 2016; Naowaboot et al., 2016). Several studies in animal models have demonstrated that the administration of morin significantly prevented liver injury, inflammation, and fibrosis (Rizvi et al., 2015; Gu et al., 2017; Perumal et al., 2017; Tian et al., 2017; Li et al., 2018). For example, MH attenuated acetaminophen-induced liver injury by potentiating Nrf2 regulated survival mechanism (Rizvi et al., 2015). Wang et al. reported that morin reduced hepatic inflammation-associated lipid accumulation in high fructose-fed rats *via* inhibition of sphingosine kinase 1/sphingosine 1-phosphate signaling pathway (Wang et al., 2013). A recent study by Tian et al. demonstrated that MH could protect LPS/d-GalN-induced acute liver injury by activating Nrf2 signal pathways and inhibiting NF-κB activation (Tian et al., 2017). But the molecular mechanism of MH for hepatoprotective effects remains to be further clarified.

In the present study, we found that acute CCl_4_ intoxication induced hepatic injury, which was manifested in increased serum markers of liver damage, hepatocyte death, macrophage infiltration, and histopathological alterations (**Figure 1**). However, MH pretreatment reduced the levels of serum ALT and AST induced by CCl_4_ (**Figure 1C**) and preserved the structural integrity of the hepatocellular membrane (**Figure 1D**), which was further supported by the histopathological findings (**Figure 1E**).

It is now generally accepted that innate immune cells, that is, infiltrating monocytes/macrophages, Kupffer cells, and neutrophils, are activated after acute liver cell death (Antoniades et al., 2008). Indeed, our result demonstrated that infiltration of the liver macrophages significantly increased to 44.97 ± 0.76 cells per HPF at 24 h after CCl_4_ injection than in normal control animals (12.83 ± 1.04 cells per HPF). But MH pretreatment in CCl_4_-injected mice remarkably diminished macrophage infiltration into the liver as determined histologically by F4/80 staining (**Figures 1F, G**). Moreover, previous studies have confirmed that TREM-1 is an activating receptor that amplifies inflammatory response and is highly expressed in neutrophils and monocytes/macrophages (Pelham and Agrawal, 2014; Tammaro et al., 2017; Tornai et al., 2019). Here, we found that TREM-1 was also involved in acute liver injury induced by CCl_4_ and that TREM-1 predominantly localized to sinusoidal regions in the liver (**Figure 2A**). In contrast, 24 h after CCl_4_ injection, the amount of TREM-1 was enhanced in the sinusoidal areas of injury livers (**Figure 2A**). However, pretreatment with MH inhibited TREM-1 expression in both gene and protein levels (**Figure 2**). Furthermore, *in vitro* LPS-stimulated RAW267.4 cells induced TREM-1 expression, while MH treatment significantly inhibited TREM-1 expression and the production of TNF-α and IL-1β (**Figures 5A, B**). Of note, this inhibitory effect MH on the expression of the TREM-1 in cell culture was further heightened after TREM-1 knockdown with siRNA (**Figure 7**). Our results and other recent reports have demonstrated that TREM-1 signaling contributes to proinflammatory pathway activation in liver injury, fibrosis, alcoholic liver disease, and NAFLD (Nguyen-Lefebvre et al., 2018; Rao et al., 2019; Tornai et al., 2019). Supporting our results, several studies in other organs have shown that TREM-1 blockade either by TREM-1-Fc or by a peptide attenuated the disease process (Klesney-Tait et al., 2006; Schenk et al., 2007; Pelham and Agrawal, 2014). Therefore, these data strongly suggested that TREM-1 may play a critical role in the pathogenesis of acute liver injury; and the hepatoprotective activity of MH was involved in the modulation of TREM-1 expression in macrophages.

Furthermore, it has been shown that TREM-1 can amplify TLR4-mediated as well as TLR2-mediated proinflammatory signaling (Ornatowska et al., 2007; Pelham and Agrawal, 2014; Tammaro et al., 2017). Particularly TLR4, which is mainly expressed on hepatic macrophages in the liver, contributes to liver injury, inflammation, and fibrosis (Mencin et al., 2009; Li et al., 2016a; Li et al., 2016b; Ding et al., 2018). In our current study, we found that pretreatment with MH of acute liver injury in mice decreased the levels of TLR4 mRNA and protein expression in livers (**Figure 3**); and this decrease may be the effect and mechanism of MH on amelioration of liver inflammation and necrosis. Consistent with our results, several *in vivo* and *in vitro* studies have also confirmed that morin effectively downregulated the expression of TLR4 (Heeba and Mahmoud, 2014; Tian et al., 2017). Additionally, blockade or silencing of TREM-1 can inhibit TLR4-mediated induction of cytokines upon LPS activation (Pelham and Agrawal, 2014; Tornai et al., 2019).

Our study has also shown that MH pretreatment potently inhibited IκBα degradation and subsequent NF-κB p65 nuclear translocation in liver of a CCl_4_-induced animal model as compared with vehicle pretreatment of mice (**Figures 4A–C**). Additionally, MH exerted an inhibitory effect on NF-κB activation and further reduced the expression of its downstream inflammatory cytokines, such as TNF-α, IL-1β, and IL-6 (**Figure 4D**). In this *in vitro* study, we also demonstrated that MH prevents the release of proinflammatory cytokines in LPS-stimulated macrophages by inhibiting TREM-1/TLR4/NF-κB signaling pathway (**Figure 6**). Similarly, it has been shown that MH suppresses the activation of NF-κB and downstream proinflammatory mediators in various experimental models (Wang et al., 2013; Tian et al., 2017; Sharma et al., 2018).

On the other hand, pretreatment with MH also obviously decreased hepatic MDA content and enhanced hepatic GSH and SOD activities that were altered by injection of a single dose of CCl_4_ (**Figures 8A–C**). GSH is a particularly efficient intracellular antioxidant that maintains intracellular redox status (McGregor and Lang, 1996), and SOD is an endogenous enzymatic antioxidant that plays synergistic actions in scavenging of free radicals by transforming them to less deleterious molecules (McGregor and Lang, 1996). Furthermore, Nrf2 is a redox-regulated transcription factor involved in the modulation of antioxidant defense systems (Anuranjani and Bala, 2014). We found that the mice receiving CCl_4_ alone have a remarkable decrease in Nrf2 protein levels in both nuclear and cytoplasm, which was reversed by MH (**Figures 8D**, **E**), suggesting that MH pretreatment to CCl_4_-induced mice significantly enhanced nuclear translocation of Nrf2 (Tian et al., 2017). Also, MH induced the expression of Nrf2’s downstream target HO-1 expression in both mRNA and protein (**Figures 8F, G**), therefore leading to enhanced antioxidative defense in the liver.

Additionally, previous studies have revealed that oxidative stress can promote inflammation by increasing NF-κB activation (Shi et al., 2003; Nery-Flores et al., 2018; Sharma et al., 2018) and that Nrf2 can negatively regulate the NF-κB signaling and can inhibit its downstream proinflammatory response and oxidative injury in mice (Shi et al., 2003; Lee et al., 2014; Wardyn et al., 2015). Thus, the inhibition of NF-κB signaling observed in the liver may be due to, in part at least, the antioxidative potency of MH.

## Conclusions

In summary, our current *in vitro* and *in vivo* study demonstrated the protective effects of MH on acute liver injury and provided a deep understanding of the molecular mechanisms of MH for hepatoprotective activity in the following ways: (i) blocking the TREM-1-mediated inflammatory response in macrophages and (ii) attenuating extensive oxidative stress through modulation of the Nrf2/HO-1 pathway in the liver. This multiple-target approach provided a potential therapeutic option to protect against liver injury.

## Data Availability

The raw data supporting the conclusions of this manuscript will be made available by the authors, without undue reservation, to any qualified researcher.

## Ethics Statement

The animal study was reviewed and approved by the Animal Care Committee of Zhongshan Hospital and the study was performed in accordance with the Guiding Principles for the Care and Use of Laboratory Animals approved by the Fudan University Animal Care Committee.

## Author Contributions

XL and CT conceived the study and wrote the manuscript; XL, QY, and CT contributed to the work designing, performing, analyzing, and interpreting data from all the experiments; QY, JH, QJ, and XL participated in the design, acquisition, analysis, and interpretation of data; CT, BX, FC, and XL carried out the animal model and all the *in vivo* animal experiments; CT and XL interpreted the data and finalized the article. All authors have critically revised and approved the final manuscript and agreed to be accountable for all aspects of the work.

## Funding

This work was supported by the National Natural Science Foundation of China (grant number: 81170398) and Shanghai Natural Science Foundation (grant number: 18ZR1406600).

## Conflict of Interest Statement

The authors declare that the research was conducted in the absence of any commercial or financial relationships that could be construed as a potential conflict of interest.
